# The integrated clinico-scientific system for gastrointestinal diseases combining Chinese and Western medicine

**DOI:** 10.1186/s12967-026-08527-x

**Published:** 2026-06-25

**Authors:** Zicheng Liang, Haorui Zha, Fengyong Wang, Wenfa Lin, Wei Zhang

**Affiliations:** 1 The Second School of Clinical Medical, Zhejiang Chinese Medical University, Hangzhou, 310005 China; 2 The Second Affiliated Hospital of Zhejiang Chinese Medical University (Xinhua Hospital of Zhejiang Province), Hangzhou, 310005 China; 3 Zhejiang-Belgium Joint Laboratory on Integrative Chinese and Western Medicine for Inflammation-to-Cancer Transformation in Gastrointestinal Diseases, Hangzhou, 310005 China; 4 The Clinico-Scientific Integration Laboratory of Integrated Chinese and Western Medicine for the Prevention and Treatment of Gastrointestinal Tumors in Zhejiang Chinese Medical University, Hangzhou, 310005 China; 5 Institute of Digestive Diseases, The Second Affiliated Hospital of Zhejiang Chinese Medical University (Xinhua Hospital of Zhejiang Province), Hangzhou, 310005 China; 6 Key Laboratory for Research On the Pathogenesis of ‘Inflammation-Cancer Transformation’ in Intestinal Diseases, Hangzhou, 310005 China

**To the Editor,**

Improving early detection, treatment precision, and long-term outcomes in gastrointestinal diseases such as colorectal neoplasia remains challenging, partly due to immune evasion, therapeutic resistance, and recurrence [1, 2]. These limitations underscore a persistent gap between mechanistic insight and effective clinical implementation, highlighting the need for approaches that more tightly integrate clinical practice with translational research [3, 4].

We describe an integrated clinico-scientific framework that combines Western medicine with traditional Chinese medicine (TCM), structured around a bidirectional loop between the clinic and research. In this model, clinical observations inform research priorities, while research findings are iteratively translated into clinical strategies. Western medicine provides the primary diagnostic and therapeutic basis, including minimally invasive surgery, enhanced recovery pathways, and systemic therapies. TCM is incorporated across the disease trajectory, particularly in perioperative and longitudinal management, with the aim of modulating treatment-related toxicity, improving functional recovery, and supporting patient-reported outcomes.

Institutional platforms enable this iterative exchange between clinical and laboratory settings (Fig. 1). As an example, a “Five-For-One” model for gastric cancer integrates multidisciplinary care, minimally invasive techniques, enhanced recovery strategies, systemic therapy, and integrated Chinese-Western medicine [5]. This approach aligns management across the disease continuum and may support more coordinated clinical decision making. Although further evaluation is required, such frameworks may provide a practical strategy for strengthening translational pathways and improving patient-centred care in complex gastrointestinal diseases. Further prospective evaluation, standardized outcome assessment, and external validation are needed to determine the broader applicability and clinical impact of this framework.

**Fig. 1:**
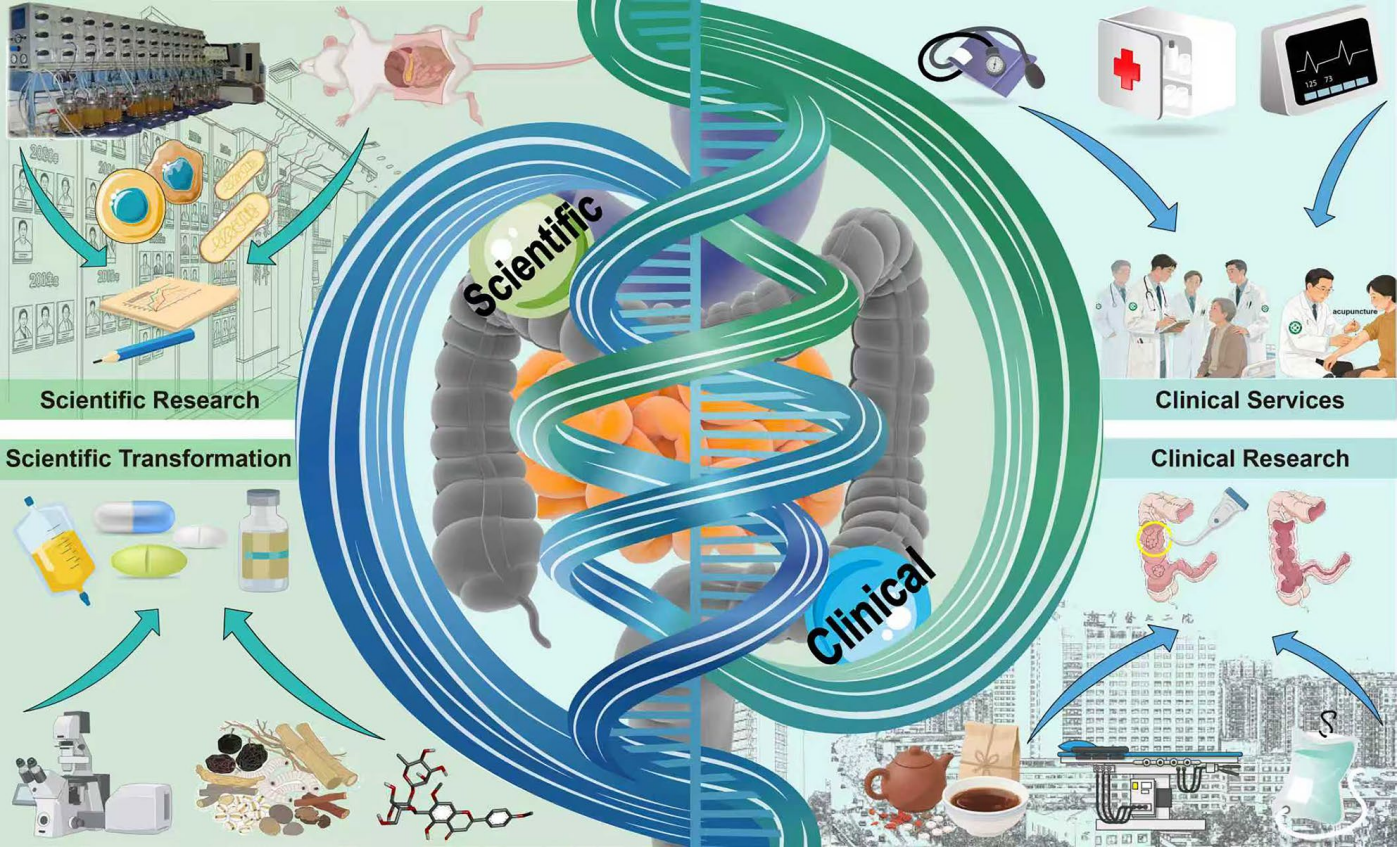
Conceptual framework of the integrated clinico-scientific system for gastrointestinal diseases combining Western medicine and traditional Chinese medicine

## Data Availability

Data sharing is not applicable to this article as no datasets were generated or analysed during the current study.
